# Influence of DNA methylation and chromatin accessibility on regulation of gene expression during *Trichomonas vaginalis-*host cell interaction

**DOI:** 10.1128/mbio.03175-25

**Published:** 2025-12-03

**Authors:** Daniela Muñoz, Ayelén Lizarraga, Patricia J. Johnson, Pablo H. Strobl-Mazzulla, Natalia de Miguel

**Affiliations:** 1 Laboratorio de Parásitos Anaerobios, Instituto Tecnológico Chascomús (INTECH), CONICET-UNSAM 591773, Chascomús, Argentina; 2 Escuela de Bio y Nanotecnologías (UNSAM), Chascomús, Argentina; 3 Department of Microbiology, Immunology & Molecular Genetics, University of California 158082, Los Angeles, California, USA; 4 Laboratorio de Biología del Desarrollo, Instituto Tecnológico Chascomús (INTECH), CONICET-UNSAM 591773, Chascomús, Argentina; University of Wisconsin-Madison, Madison, Wisconsin, USA

**Keywords:** *Trichomonas vaginalis*, DNA methylation, epigenetics, chromatin, host-cell interactions

## Abstract

*Trichomonas vaginalis* is a common sexually transmitted parasite that colonizes the human urogenital tract, causing infections that range from asymptomatic to highly inflammatory. As an extracellular pathogen, adherence to host epithelial cells is an important step to colonize the human host. Hence, understanding how the process of attachment to host cells is regulated remains an important goal in parasitology research and human health. *T. vaginalis*-host interaction is regulated by changes in gene expression, but it is still largely unknown how these changes in transcriptional profiles are controlled, as very few transcriptional regulatory elements have been described. Our recent work highlighted the importance of epigenetics in the regulation of transcription, and a specific role for N6-methyladenine (6mA) in modulating three-dimensional chromatin structure has been suggested. Building on these findings, we analyzed here the role of 6mA and chromatin accessibility during the process of host-parasite interaction by integrating MeDIP-seq and assay for transposase-accessible chromatin sequencing data with RNA-seq in free vs host cell-attached parasites. Consistent with our previous results, we identified transcriptionally active and repressive regions flanked by 6mA modifications, observed both in the presence and absence of host cells. Importantly, we detected differentially accessible chromatin regions that influence the gene expression of key pathogenesis-related genes during *T. vaginalis* host cell interaction. These findings highlight the importance of chromatin architecture in regulating gene expression during parasitic infection.

**IMPORTANCE:**

*Trichomonas vaginalis*, the most common non-viral sexually transmitted parasite, relies on adherence to host epithelial cells to establish infection. Our previous work highlighted the importance of N6-methyladenine (6mA) DNA methylation in the regulation of transcription and three-dimensional chromatin structure. Now, our study integrates RNA-seq, MeDIP-seq, and assay for transposase-accessible chromatin sequencing data to reveal how 6mA and chromatin accessibility modulate gene expression during *T. vaginalis* interaction with human host cells. We identified over 3,600 differentially expressed genes upon parasite contact with prostate cells, including pathogenesis-related genes. Moreover, we identified transcriptionally active and repressive regions flanked by 6mA that remain largely stable during the process of host interaction. We mapped genome-wide chromatin accessibility and uncovered differentially accessible regions upon host cell contact associated with a subset of genes involved in adhesion. These results suggest that local chromatin accessibility has a major role in modulating gene expression of key virulence genes during host interaction.

## INTRODUCTION

*Trichomonas vaginalis* is a unicellular, extracellular, flagellated protozoan that causes trichomoniasis, the most frequent non-viral sexually transmitted infection globally, with a total of ~156 million reported infections annually (1). This parasite colonizes the human urogenital tract, causing a generally mild or asymptomatic infection. When symptoms do occur, disease manifestation might include discomfort in the urogenital area as a consequence of vaginitis or urethritis (2). Importantly, chronic infections have been associated with serious complications such as infertility, premature birth, increased HIV susceptibility, cervical cancer, and aggressive prostate cancer (3–7).

In order to successfully establish and maintain infection, parasites must rapidly and efficiently adapt to different environments to colonize their hosts (8, 9). As an exclusively extracellular pathogen, *T. vaginalis* adheres to the epithelial lining of the urogenital tract by transitioning from a free-swimming ovoid cell into its adherent amoeboid form to establish infection (10, 11). Parasite adherence to the host epithelium is a multifactorial process that involves different factors such as lipoglycans (12, 13), extracellular vesicles (14–18), BspA-like proteins (19, 20), Bap-like proteins (19, 21), a rhomboid serine protease (22), tetraspanin proteins (15, 23), VPS32 (member of the ESCRT-III complex) (14), and a host glycosaminoglycan-interacting protein (24), among other factors. While no individual player can fully recapitulate maximum adherence, different studies reveal that the overexpression of specific genes can enhance parasite attachment (14, 19, 20, 23, 25). This underscores the significance of precise regulation of gene expression levels of adherence factors in regulating parasite adherence. In concordance, several studies have reported drastic changes in specific genes in the transcriptional repertoire that occur when parasites are exposed to host cells or adverse growing conditions (26, 27). However, the mechanisms involved in transcriptional regulation, critical for modulating the infection process, remain poorly understood. Only a few core promoter elements or transcription factors have been identified to date (28), and no other mechanisms regulating gene expression have been fully characterized. However, recent studies indicate that epigenetics has an important role in controlling transcription in *T. vaginalis* (29). Specifically, histone acetylation (H3KAc) has been identified as a permissive histone modification that functions to mediate chromatin accessibility, gene transcription, and pathogenesis in *T. vaginalis* (21, 30). The DNA methylation mark N6-methyladenine (6mA) has emerged as a significant epigenetic modification potentially influencing three-dimensional (3D) genome organization and gene expression. In this sense, three-dimensional arrangements of DNA in the nucleus are increasingly seen as key contributors to the regulation of gene expression, but studies on how genome structure and nuclear organization influence transcription have so far been limited to a handful of model species. In particular, chromatin looping is a type of intrachromosomal interaction that has been shown to influence gene expression (31, 32). In *T. vaginalis*, bioinformatics analyses revealed clusters of transcriptionally active or repressive genes flanked by intergenic regions enriched in 6mA (33). Chromatin conformation capture experiments showed that some of these 6mA-enriched flanking regions are in close spatial proximity, suggesting the formation of chromatin loops (33). As a step toward understanding the role of three-dimensional chromatin architecture and 6mA DNA methylation during *T. vaginalis*-host cell interaction, we examined gene expression changes potentially modulated by 6mA methylation and/or chromatin accessibility. While most of the genes with detected reads were unchanged (20,884 genes), we identified 3,623 differentially expressed genes (DEGs) when *T. vaginalis* was exposed to prostate cells, including 2,069 upregulated and 1,554 downregulated genes. Approximately 4,300 transcriptionally active or repressive regions flanked by 6mA modifications were detected both in the presence and absence of host cells, with no major changes in their distribution following host exposure. However, we observed differentially accessible chromatin regions associated with the regulation of key pathogenesis-related genes during host interaction. Overall, our findings suggest that chromatin accessibility plays a significant role in modulating gene expression during *T. vaginalis* infection.

## RESULTS

### Identification of genes differentially expressed during the process of host-parasite interaction

**Fig 1:**
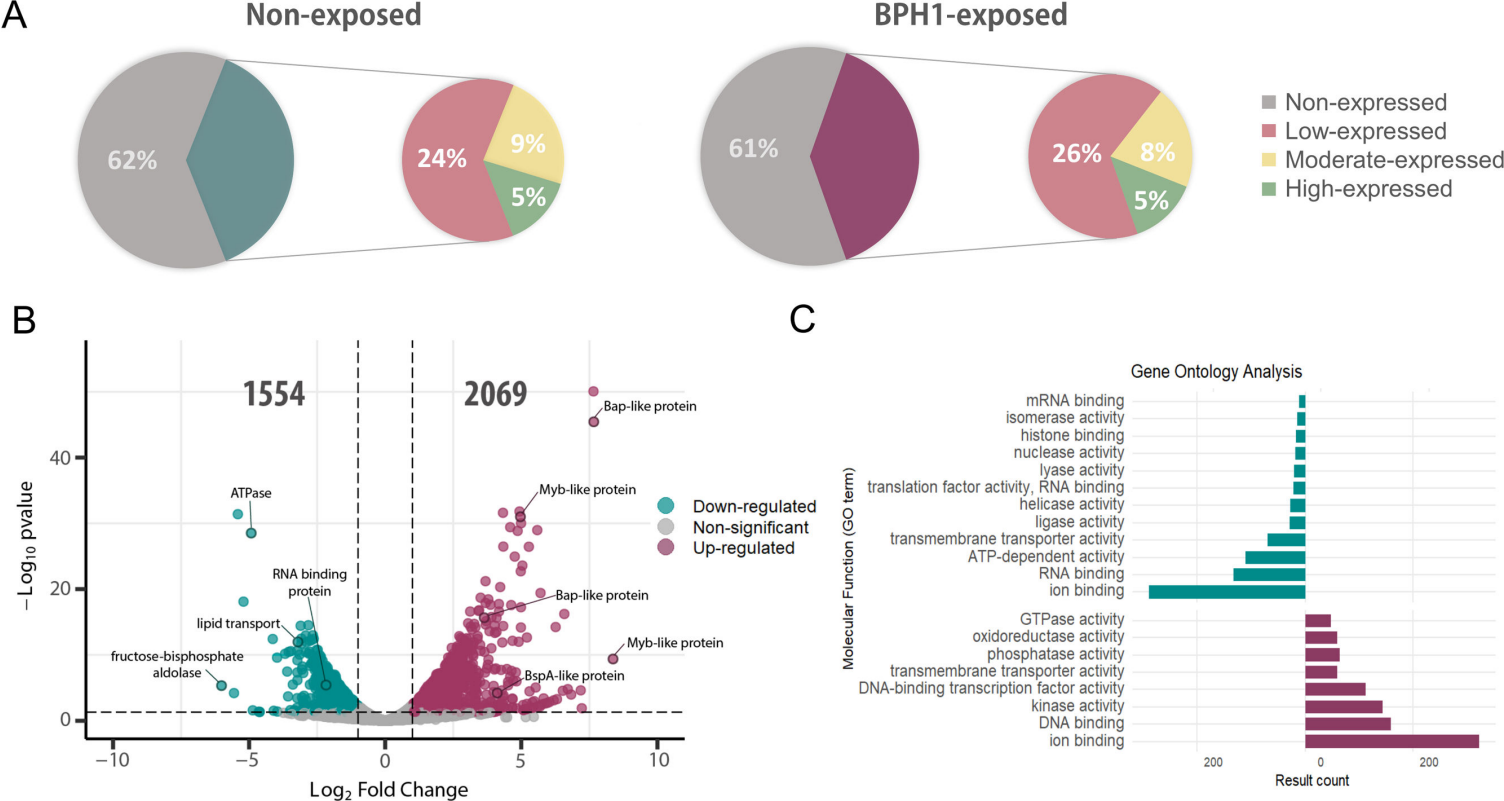
Transcriptomic changes in *Trichomonas vaginalis* during interaction with BPH1 cells. (**A**) Genes were categorized based on their expression levels in non-exposed and BPH1-exposed parasites. The percentage of non-expressed (gray), low-expressed (red), moderate-expressed (yellow), and high-expressed (green) genes in non-exposed and BPH1-exposed parasites is shown. (**B**) Volcano plot showing differential gene expression between non-exposed and BPH1-exposed parasites. Significantly upregulated genes upon exposure to host cells are highlighted in magenta, while significantly downregulated genes are shown in cyan (|log2 fold change| ≥ 1 and *P*-value < 0.05). (**C**) GO analysis of differentially expressed genes, categorized by molecular function. Bars represent the number of genes associated with each GO term for upregulated (magenta) and downregulated (cyan) genes upon exposure to host cells.

To assess gene expression changes during *T. vaginalis* interaction with host cells, we conducted RNA-seq analysis comparing gene expression levels in the free-living parasites and parasites exposed to prostate cells (B7268 strain). Specifically, parasites from the highly adherent B7268 strain (34) were exposed to adult, non-tumorigenic, spontaneously immortalized human prostate epithelial cell lines (BPH1) for 90 min (35), and the level of gene expression in BPH1-exposed parasites was compared to non-exposed parasites. Out of the 88,188 genes identified in the latest reference genome annotation (36), approximately 60% were non-expressed under either of the analyzed conditions (Fig. 1A). Upon analyzing the transcriptional levels of expressed genes, approximately 25% exhibited low expression (FPKM > 0 and ≤ 5), around 9% showed moderate expression (FPKM > 5 and ≤ 20), and only 5% displayed high expression (FPKM > 20) under both analyzed conditions (Fig. 1A). Principal component analysis (PCA) reveals that transcriptomes from replicates are more similar to each other than those from different treatments (PC1, 84% and PC2, 11% variance) (Fig. S1A). While most of the genes with detected reads were unchanged (20,884 genes), we identified 3,623 DEGs when *T. vaginalis* was exposed to BPH1 cells, including 2,069 upregulated and 1,554 downregulated genes (Fig. 1B). Next, we analyzed the enriched molecular function of DEGs in *T. vaginalis* upon exposure to host cells using gene ontology (GO) analysis (Fig. 1C). Our results showed that genes upregulated in the highly adherent B7268 strain in contact with host cells were associated with DNA binding and transcription factor activity, kinase activity, and phosphatase activity, among others (Fig. 1C). Many of the upregulated genes belonged to protein families previously linked to parasite pathogenesis (Table S1). These included approximately 60 leucine-rich repeat BspA family genes, 16 Bap-like genes, 8 cysteine-type proteases, 11 serine-type endopeptidases, and 87 serine/threonine kinases (Table S1). Similar to previous findings when *T. vaginalis* was exposed to vaginal epithelial cells (26), we have also identified upregulated gene families including MYB family transcription factors, DNA- and RNA-binding proteins, and a diverse range of kinases as well as calmodulin, ubiquitin-conjugating enzymes, and many RNA polymerase II transcription regulator-recruiting proteins (Table S1). Alternatively, the enriched molecular function associated with downregulated genes was related to ion and RNA binding, ATP-dependent and transporter activity, among others (Fig. 1C). Collectively, these data reveal a diverse set of differentially expressed genes that may contribute, at least in part, to the process of parasite interaction with host cells by modulating the initial adherence and/or the maintenance of attachment.

### Distribution of 6mA DNA methylation during host-parasite interaction

**Fig 2:**
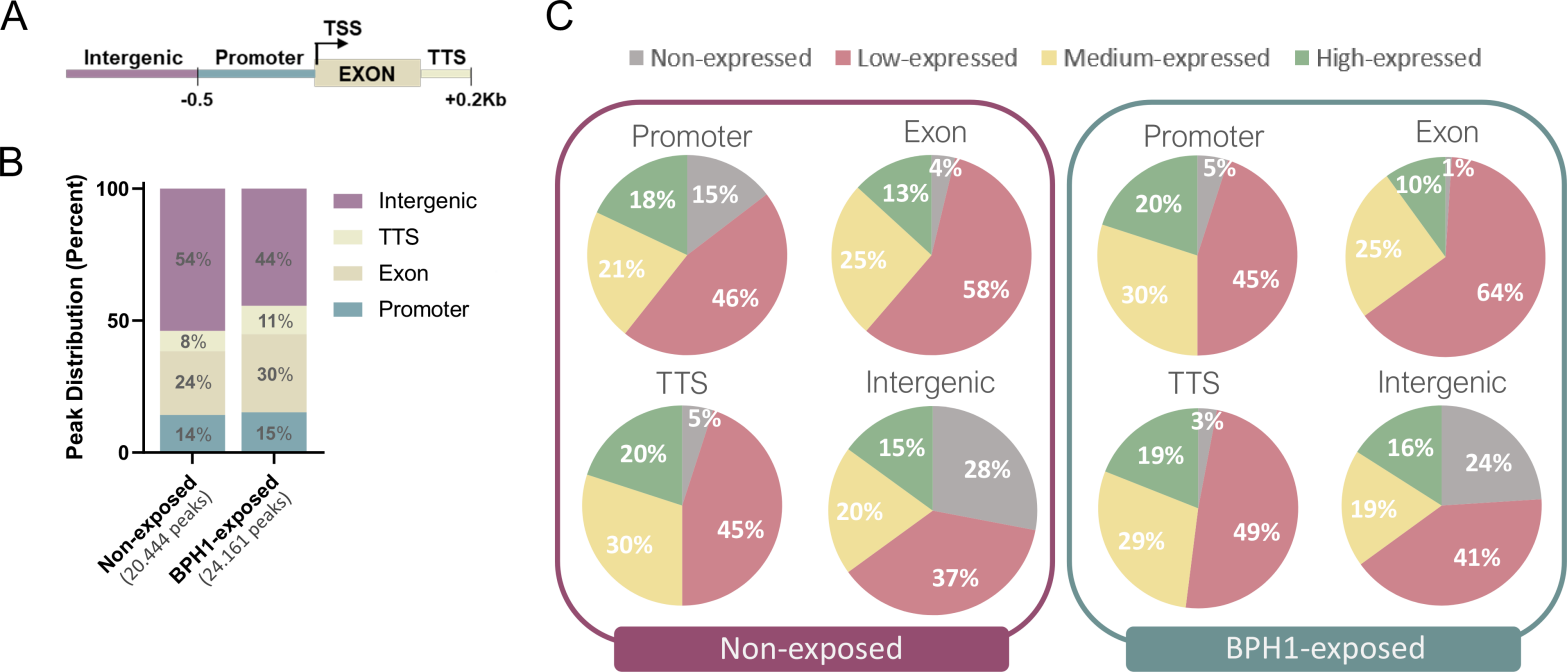
Distribution of 6mA peaks across genomic regions and their association with gene expression levels. (**A**) Schematic representation of genomic features analyzed: intergenic regions, promoters (±500 bp from TSS), exons, and TTS (±200 bp from TTS). (**B**) Bar graph showing the percentage of MeDIP-seq peaks distributed across genomic features in non-exposed and BPH1-exposed *Trichomonas vaginalis*. Total number of peaks identified in each condition is indicated above the bars. (**C**) Pie charts representing the expression levels of genes (non-, low-, medium-, and high-expressed) associated with detected 6mA peaks across promoters, exons, TTS, and intergenic in non-exposed (left) and BPH1-exposed (right) parasites.

**Fig 3:**
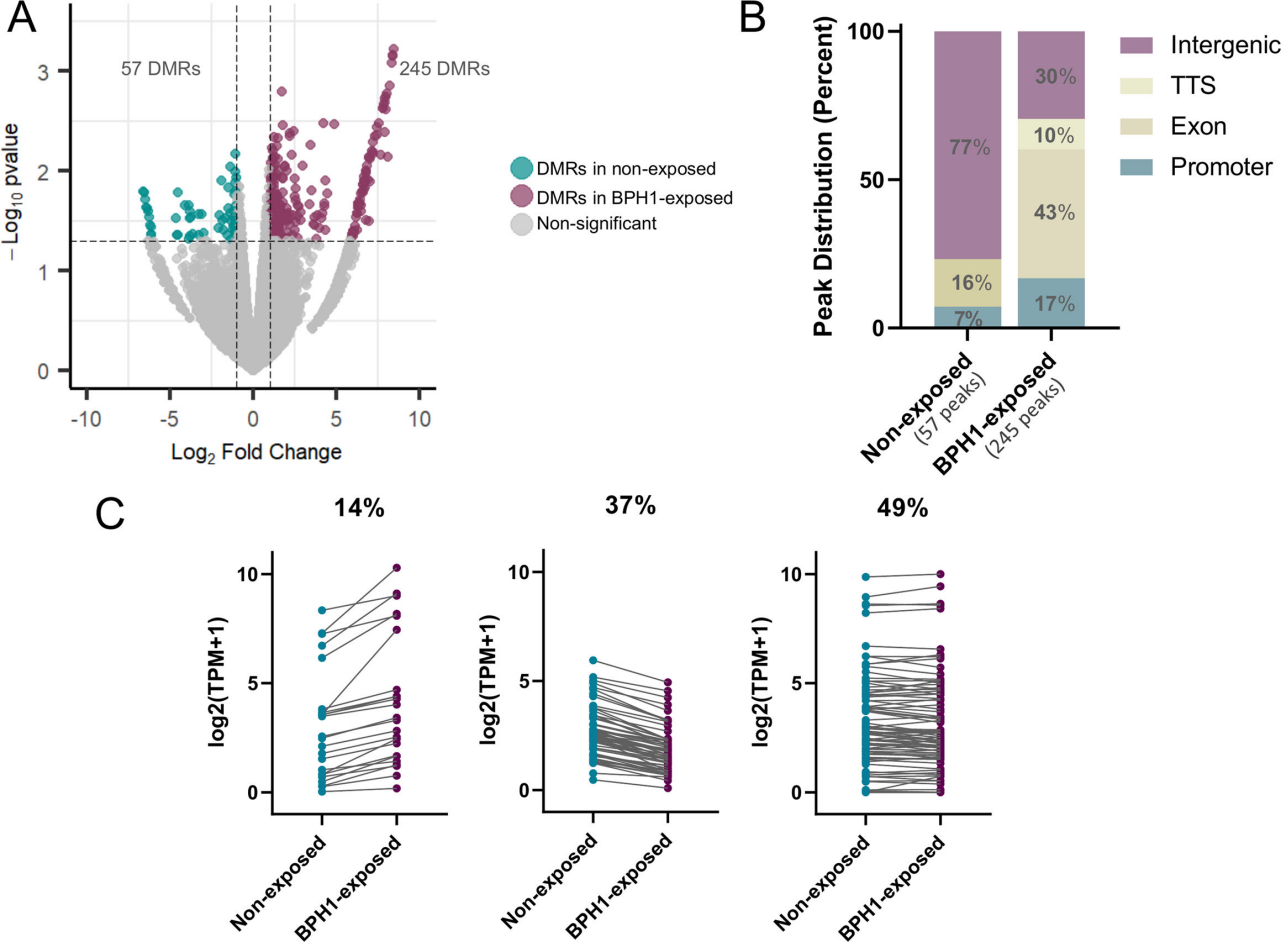
DMRs between non-exposed and BPH1-exposed parasites. (**A**) Volcano plot showing differentially methylated regions between non-exposed and BPH1-exposed parasites. Significantly hyper-methylated regions in BPH1-exposed parasites are highlighted in magenta, non-significant are shown in gray, and hyper-methylated in non-exposed parasites are highlighted in cyan (|log2 fold change| ≥ 1 and *P*-value < 0.05). (**B**) Bar graph showing the genomic distribution of differentially methylated regions distributed across genomic features. (**C**) Expression levels (log2 TPM + 1) of genes linked to DMRs located in promoters, exons, or TTS were compared between non-exposed and BPH1-exposed parasites. Genes were classified based on the ratio of expression (TPM) between conditions as increased (≤0.75), reduced (≥ 1.5), or unchanged (0.75–1.5). Each panel shows one category, with blue and purple dots representing expression in non-exposed and BPH1-exposed parasites, respectively. Lines connect the same gene in both conditions. The percentage on top of each panel indicates the proportion of genes in that category.

To investigate the role of DNA methylation in parasite-host interaction, we analyzed changes in the distribution and density of 6mA throughout the genome of parasites exposed to BPH1 cells compared to those not exposed. To this end, we performed a MeDIP-seq assay using a 6mA-specific antibody to enrich for DNA fragments containing 6mA from both free-living B7268 parasites and those exposed to BPH1 cells for 90 min. After quality filtering (*q* value < 0.01, fold enrichment > 2), iterative overlap peak merging was performed, and a total of 20,444 6mA peaks were obtained from samples of non-exposed parasites and 24,161 peaks from parasites exposed to BPH1 cells (Fig. 2B; Table S2). PCA reveals that the methylation profile in replicates is more similar to each other than to those between treatments (PC1, 76% and PC2, 17% variance) (Fig. S1B). To assess potential enrichment or depletion of 6mA in specific genomic features, we analyzed the distribution of peaks across various genomic regions. To this end, we defined four regions of interest: coding regions (exon), intergenic regions, proximal promoters, defined as ±500 bp from the transcription start site (TSS) of each annotated gene, and transcription termination site (TTS), defined as ±200 bp from the TTS of each annotated gene (Fig. 2A). The distribution of 6mA peaks across different genomic regions was similar between samples of BPH1-exposed and non-exposed parasites (Fig. 2B). The analysis of genomic distribution in free-living parasites revealed that 14% of the detected 6mA peaks were located in promoter regions, 24% were found within exons, while intergenic regions and TTSs accounted for 54% and 8%, respectively (Fig. 2B). In parasites exposed to BPH1 cells, 15% of 6mA peaks covered promoters, 30% were found in exons, while intergenic regions and TTS accounted for 44% and 11%, respectively (Fig. 2B). To evaluate the influence of 6mA in controlling the level of gene expression in *T. vaginalis*, we performed a combined analysis of RNA-seq and MeDIP-seq data in samples from free-living parasites and those exposed to BPH1 cells. The analysis showed that when 6mA is located in promoters, exons, or TTS regions, a large proportion of the corresponding genes are either not expressed (FPKM = 0) or expressed at low levels (FPKM > 0 and ≤ 5) in both conditions analyzed (Fig. 2C). As described previously (33), a significant proportion of 6mA peaks are located within intergenic regions in both conditions (Fig. 2B). To determine whether 6mA might influence the expression of nearby genes, we analyzed the expression levels of the genes located closest to the 6mA intergenic peaks (Fig. 2C). The analysis revealed that more than 60% of the 6mA peaks have a non- or low-expressed adjacent gene in free-living and BPH1-exposed parasites (Fig. 2C), suggesting that intergenic methylation may be associated with reduced gene activity of the nearest gene. Then, we performed a differential analysis of methylated regions between free-living and BPH1-exposed parasites. By doing this analysis, 57 differentially methylated regions (DMRs) were detected in non-exposed parasites, while 245 differentially methylated regions were found in the BPH1-exposed parasites (LFC ≥ 1 and *P*-values ≤ 0.05) (Fig. 3A; Table S3). When the distribution of these DMRs was evaluated, we found that most DMRs in non-exposed parasites were located in intergenic regions (77%), with a smaller fraction in exons (16%) and promoters (7%). In contrast, the distribution changed in BPH1-exposed parasites, having 30% of DMRs in intergenic regions, 43% in exons, 10% in TTS, and 17% at promoters.

To further investigate the impact of 6mA methylation on gene expression, we focused on the intragenic DMRs (located in promoters, exons, and TTS) and examined the expression of the correspondent genes in BPH1-exposed and non-exposed parasites (Fig. 3C). Gene expression changes were classified based on TPM ratios between non-exposed and BPH1-exposed samples, defining genes as reduced if the ratio was ≥1.5, increased if ≤0.75, and unchanged otherwise. Our analysis revealed that a significant proportion of genes (49%) did not exhibit noticeable changes in expression despite being differentially methylated. Additionally, 37% of the genes associated with these DMRs exhibited a reduction in expression, supporting the idea that 6mA methylation may act as a negative regulatory mechanism in specific genomic contexts. Additionally, a smaller fraction (14%) displayed increased expression when methylated. These findings suggest that 6mA methylation may contribute to gene regulation by modulating gene expression in a context-dependent manner. Although DNA methylation might be influencing gene expression, it is unlikely to be a pivotal factor in transcriptional regulation during host adherence. Our results, instead, highlight a more complex interplay of regulatory mechanisms that modulate gene expression in response to host interaction.

**Fig 4:**
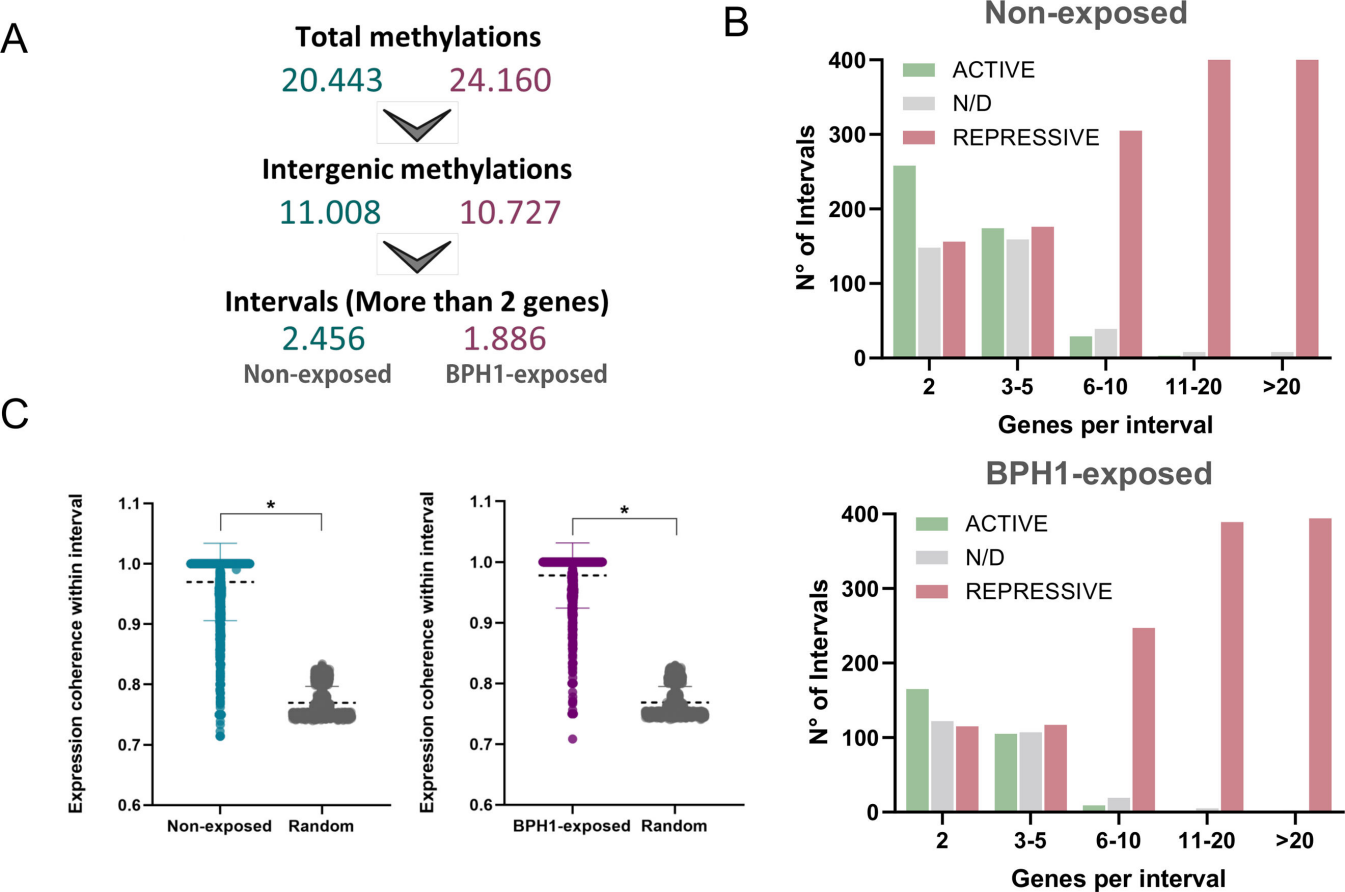
Analysis of intergenic methylation and 6mA-flanked intervals. (**A**) Workflow showing the number of 6mA-flanked intervals obtained from MeDIP-seq for non-exposed (cyan) and BPH1-exposed (magenta) conditions. (**B**) Distribution of 6mA-flanked intervals by gene number, categorized as active (green), repressive (red), or non-defined (gray), in non-exposed (top) and BPH1-exposed (bottom) conditions. (**C**) Distribution of expression coherence for genes within 6mA-flanked intervals compared to randomly selected genomic intervals of the same size, under non-exposed (left) and BPH1-exposed (right) conditions. Genes within intervals show significantly higher coherence than random genes, indicating coordinated transcriptional behavior within 6mA-defined intervals. Asterisks indicate statistically significant differences in both conditions, each with *P* < 2.2 × 10^−16^ (Wilcoxon rank-sum test).

Our previous results from chromatin conformation capture (3C) experiments indicate that intergenic 6mA might be associated with chromatin loop formation in *T. vaginalis* (33), raising the intriguing possibility that 6mA could have a role in modulating 3D genome architecture by forming transcriptionally active or repressed regions. Thus, we investigated whether 6mA-flanking regions might be linked to the formation of transcriptionally active or repressive intervals during *T. vaginalis* interactions with host cells. We defined repressive or active intervals as regions flanked by two intergenic 6mA peaks that contained two or more genes with similar level of expression. Among the total methylated regions, 11,008 intergenic peaks were identified in samples from non-exposed parasites, while 10,727 were detected in the parasites exposed to BPH1 (Fig. 4A). Among these intergenic methylated regions, we identified a total of 2,456 6mA-flanked intervals in the free-living parasites samples and 1,886 intervals in the samples corresponding to BPH1-exposed parasites (Fig. 4A; Table S4). Intervals were considered “active” if more than 70% of the contained genes had FPKM >1, “repressive” if more than 70% of genes had FPKM ≤1, and “non-defined” if none of the previous conditions were satisfied (Fig. 4B; Table S4). As previously described (33), intervals containing a greater number of genes tend to be silenced in free-living as well as parasites exposed to BPH1 cells (Fig. 4B). Conversely, most of the intervals containing fewer genes are active (Fig. 4B). To evaluate whether genes within 6mA-flanked intervals display coordinated transcription, we compared their expression coherence to randomly selected genomic intervals of the same size. Genes within 6mA-defined intervals showed significantly higher coherence than random genes in both conditions (*P*-value < 2.2 × 10^−16^, Wilcoxon rank-sum test). This coordination was further supported by intraclass correlation coefficient (ICC) analysis, which revealed high expression coherence among genes within the intervals (ICC = 0.718 for non-exposed; ICC = 0.725 for BPH1-exposed), indicating that genes within 6mA-flanked regions tend to be transcribed in a coordinated manner (Fig. 4C).

### Chromatin accessibility during parasite-host interaction

**Fig 5:**
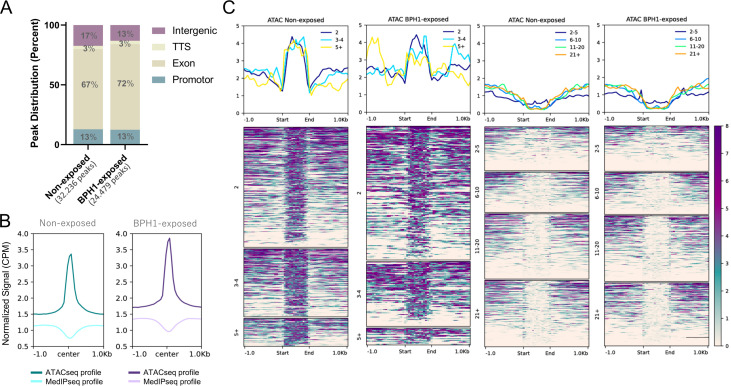
Chromatin accessibility distribution under non-exposed and BPH1-exposed conditions. (**A**) Bar graph showing the percentage of ATAC-seq peaks distributed across genomic features in non-exposed and BPH1-exposed parasites. Total number of peaks identified in each condition is indicated above the bars. (**B**) Profile plots showing the normalized signal (CPM) for ATAC-seq and MeDIP-seq peaks centered on ATAC-seq peaks in both conditions. ATAC-seq profile is shown in dark cyan (non-exposed) and dark magenta (BPH1-exposed), indicating chromatin accessibility. MeDIP-seq profile is depicted in light cyan (non-exposed) and light magenta (BPH1-exposed). (**C**) Density heatmaps and average profiles of ATAC-seq signal across active (left) and repressive (right) 6mA-flanked intervals, stratified by the number of genes per interval. Active and repressive 6mA-delimited intervals identified in non-exposed and BPH1-exposed parasites were scaled to the same size. “Start” and “End” of the intervals correspond to 6mA peak positions. Each row represents a specific interval, and color intensity corresponds to normalized read density.

Chromatin accessibility is a crucial factor in regulating gene expression. To investigate if changes in chromatin structure and accessibility might play a role during the process of host cell attachment, we performed an assay for transposase-accessible chromatin sequencing (ATAC-seq) on free-living parasites as well as parasites exposed to BPH1 cells. ATAC-seq uses a hyperactive Tn5 transposase to simultaneously cut and ligate adapters for high-throughput sequencing at regions of increased accessibility. This technique allows multidimensional assays of the regulatory landscape of chromatin, providing base-pair resolution of nucleosome-free regions in the genome (37, 38). We performed three independent ATAC-seq experiments for each condition, and the PCA profile of the data shows that the similarity between replicates is higher than the similarity between conditions (Fig. S1C). We identified 32,236 and 24,479 accessible regions in non-exposed and BPH1-exposed parasites, respectively (Fig. 5A; Table S5). We next examined the genomic distribution of open chromatin peaks across genomic elements within each condition using the reference genome annotation. We defined the same four regions of interest using the same criteria as described previously: coding regions (named exons), intergenic regions, proximal promoters, and TTS. The distribution of ATAC-seq peaks across various genomic regions was similar under both conditions analyzed, with approximately 70% of accessible chromatin located within coding sequences in both non-exposed and BPH1-exposed parasite samples (Fig. 5A).

**Fig 6:**
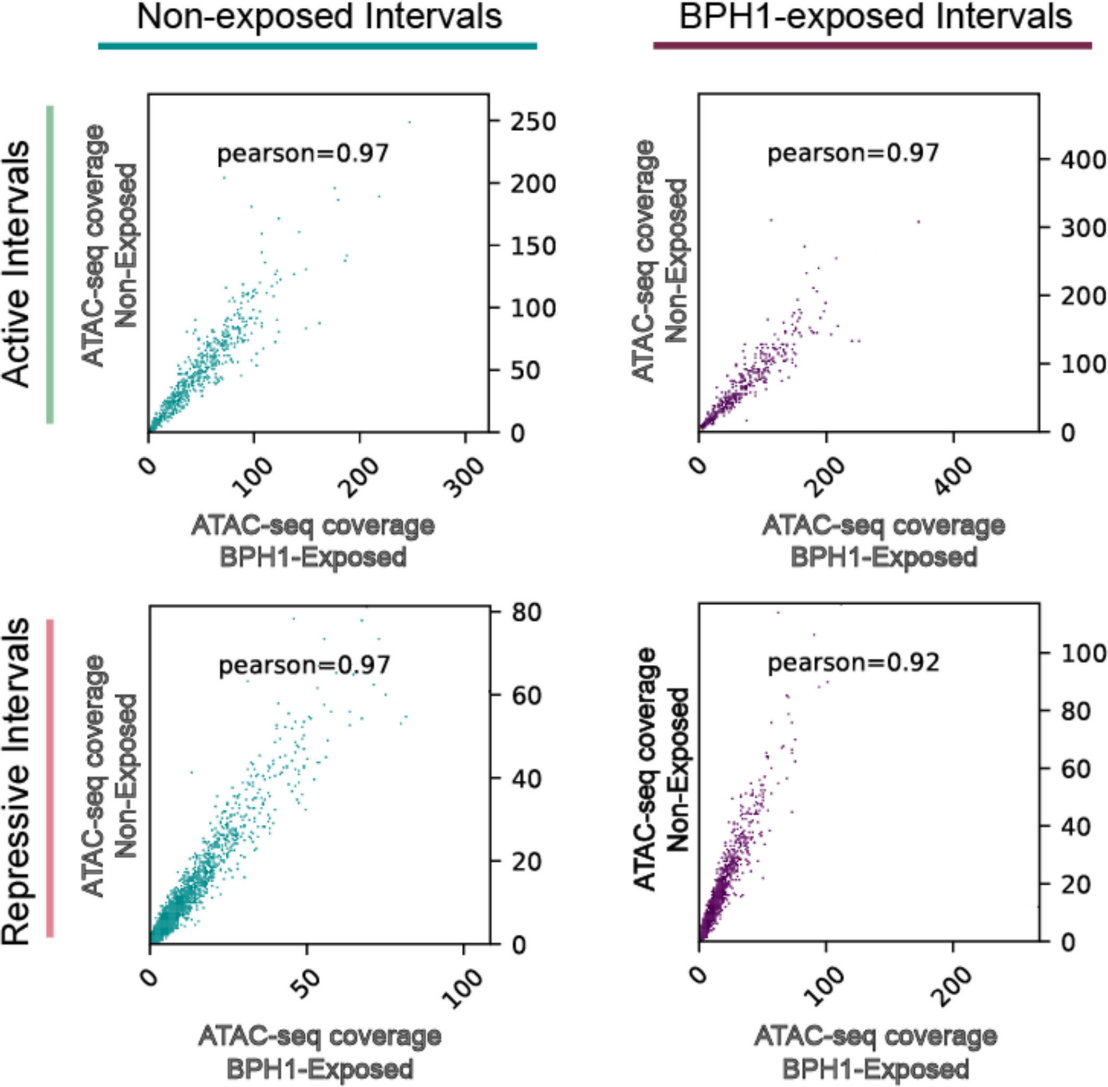
Correlation between ATAC-seq coverage within 6mA-flanked intervals in non-exposed and BPH1-exposed parasites. Scatter plots represent ATAC-seq coverage for intervals categorized as active (top panels) and repressive (bottom panels) in BPH1-exposed (*x*-axis) and non-exposed (*y*-axis) conditions. Pearson correlation coefficients are shown in each panel.

To evaluate if the level of chromatin accessibility is associated with 6mA distribution, we compared our ATAC-seq and MeDIP-seq data. Our results revealed that open chromatin regions negatively correlate with 6mA position, whether parasites are non-exposed or exposed to BPH-1 cells (Fig. 5B). These results are in concordance with our previous results, indicating that 6mA methylation at promoter or gene coding regions may be linked to reduced transcriptional activity across both conditions (Fig. 2B). We next explored the relationship between chromatin accessibility and the predicted active and repressive 6mA-delimited intervals to assess whether changes in chromatin accessibility within these regions might contribute to the coordination of transcriptional activation or repression during *T. vaginalis*-host interactions. By integrating ATAC-seq data with RNA-seq and MeDIP-seq data, we were able to define the chromatin landscape surrounding 6mA-flanked intervals. Interestingly, open chromatin regions showed a clear enrichment within predicted active 6mA-delimited intervals compared to the surrounding regions (Fig. 5C). In contrast, predicted transcriptionally repressed 6mA-delimited intervals were underrepresented in open chromatin regions (Fig. 5C). This pattern persisted regardless of the number of genes contained within the intervals (Fig. 5C). These results suggest that chromatin accessibility correlates with the expression level of genes located between the 6mA-flanked intervals. To determine whether changes in chromatin accessibility within specific intervals play a role during the process of parasite-host interaction, we analyzed the ATAC-seq coverage within the detected 6mA-delimited intervals in both non-exposed and BPH1-exposed parasite samples. Pearson correlation analysis revealed a high degree of similarity between the two conditions, with a correlation coefficient of ~0.97 for active intervals in non-exposed and BPH1-exposed samples, and ~0.97 and ~0.92 for repressive intervals in non-exposed and BPH1-exposed samples, respectively (Fig. 6). These results suggest that chromatin accessibility in these regions remains largely unaffected upon exposure of *T. vaginalis* to host cells (Fig. 6). Consistent with this, among the 245 DMRs identified between non-exposed and BPH1-exposed parasites, only 72 were located in intergenic regions, and 22 were identified at 6mA-delimited intervals (Table S3). Although DMRs were present within these 22 6mA-delimited intervals, no coordinated changes in gene expression were observed upon exposure of the parasites to host cells. Although the chromatin accessibility analysis supports the presence of 6mA-flanked intervals in both conditions, these findings suggest that chromatin intervals remain largely stable during the process of host-parasite interaction. Together, our data indicate that these intervals are unlikely to serve as the primary regulatory mechanism driving chromatin and transcriptional changes associated with parasite adherence to host cells.

### Chromatin accessibility regulates expression of pathogenesis-related genes

**Fig 7:**
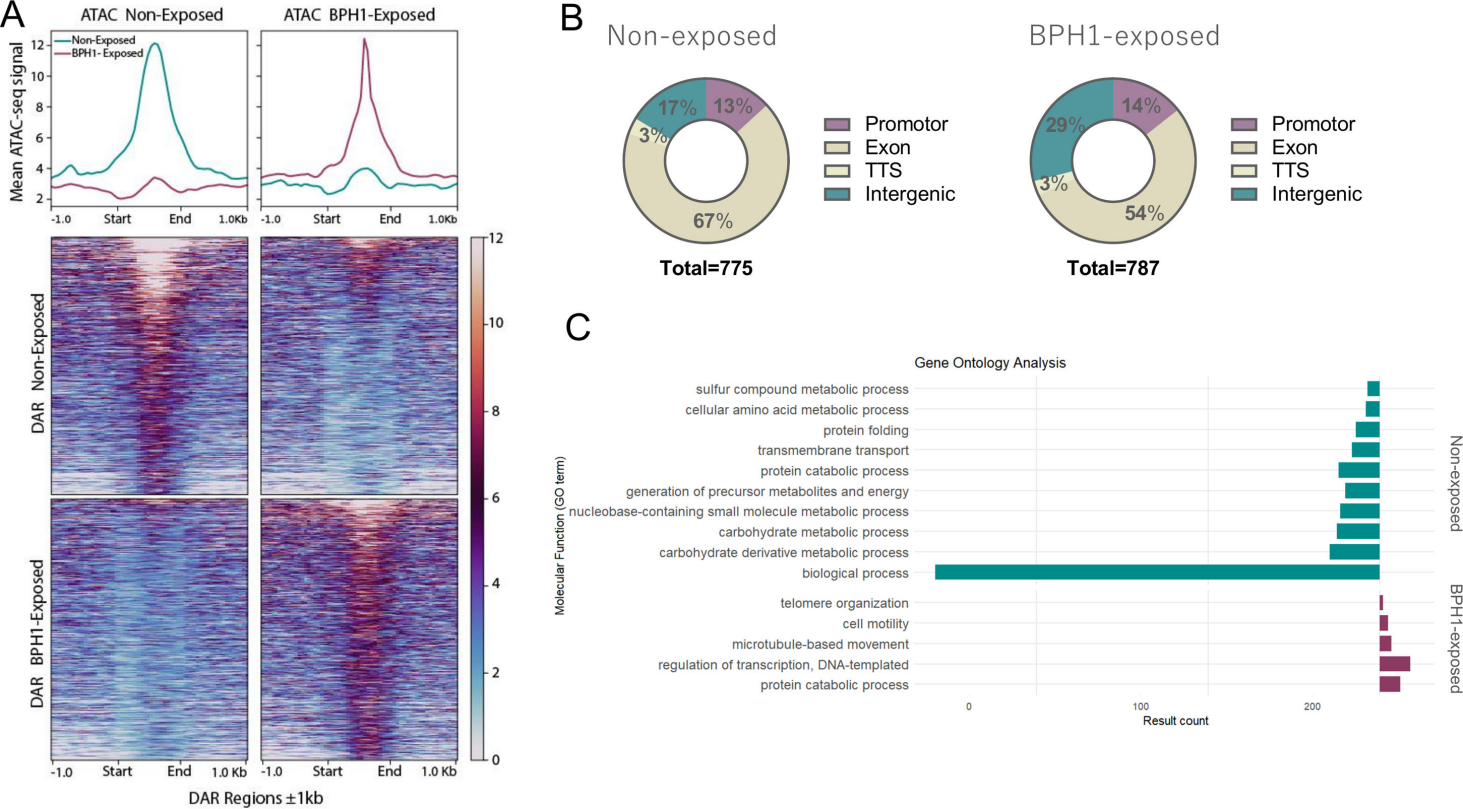
DARs detected under non-exposed and BPH1-exposed conditions. (**A**) Profiles and heatmaps of differential ATAC-seq peaks categorized by condition: non-exposed (top) and BPH1-exposed (bottom). (**B**) Genomic distribution of DARs detected at promoter, exon, TTS, and intergenic regions for non-exposed (left) and BPH1-exposed (right) conditions. The total number of differential peaks analyzed is indicated. (**C**) GO analysis of molecular functions associated with genes linked to DARs in non-exposed (cyan) and BPH1-exposed (purple) conditions.

**Fig 8:**
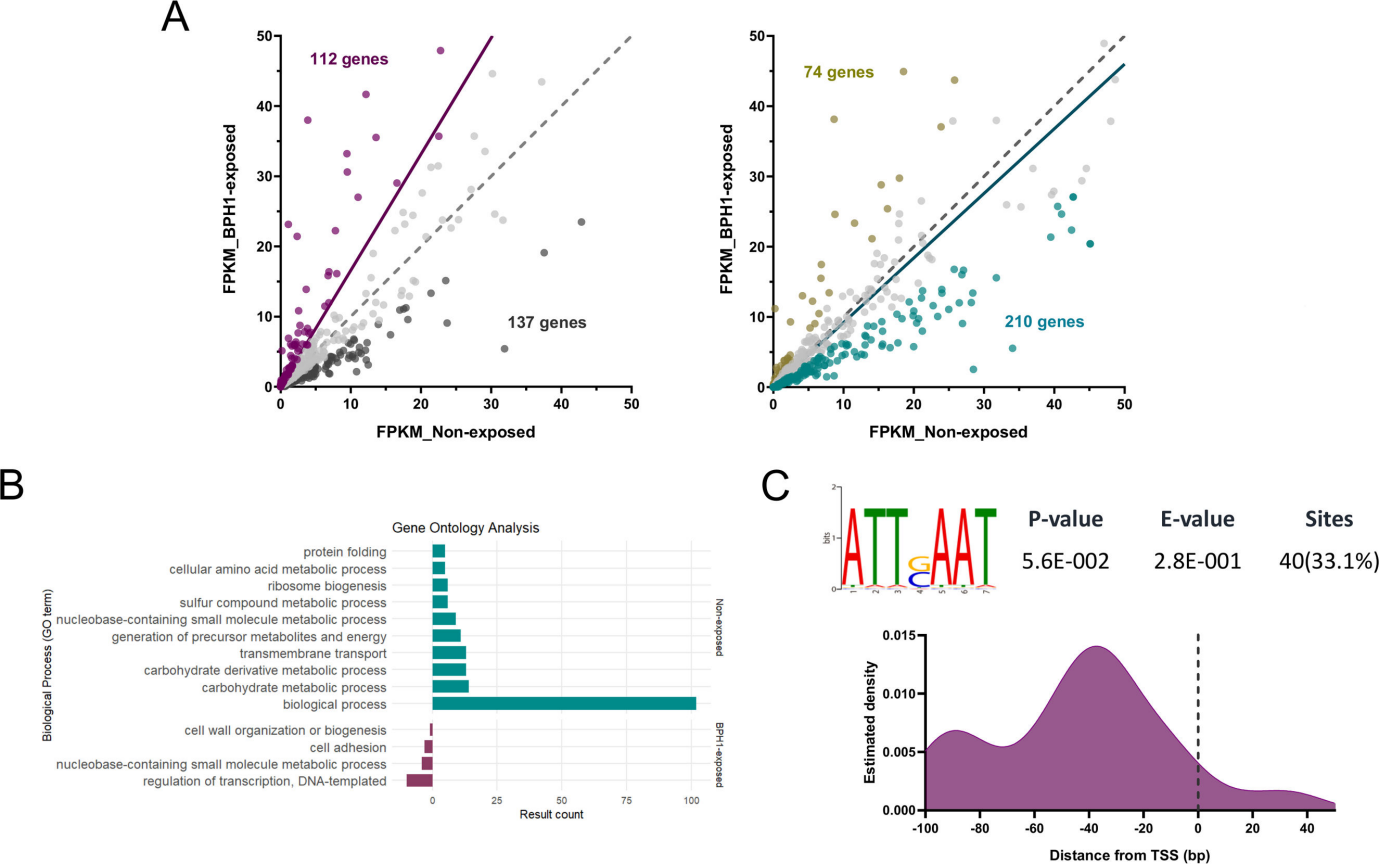
Correlation between DARs and gene expression. (**A**) Scatter plots showing the expression levels (FPKM) of genes associated with promoter/exonic DARs identified in BPH1-exposed parasites (left) and in non-exposed parasites (right). Each dot represents a gene plotted according to its FPKM values in both conditions. The dashed gray line indicates the expected distribution if expression levels were equal between conditions, while the colored line (violet for BPH1-exposed and cyan for non-exposed) shows the observed trend in each plot. (**B**) GO enrichment analysis of genes showing correlated accessibility and expression changes under each condition. (**C**) Enriched motif identified among genes with correlated DARs and expression in the BPH1-exposed condition (violet), with its genomic distribution relative to TSS. The curve represents the kernel density estimation (Gaussian kernel), calculated in R and plotted in GraphPad Prism.

**Fig 9:**
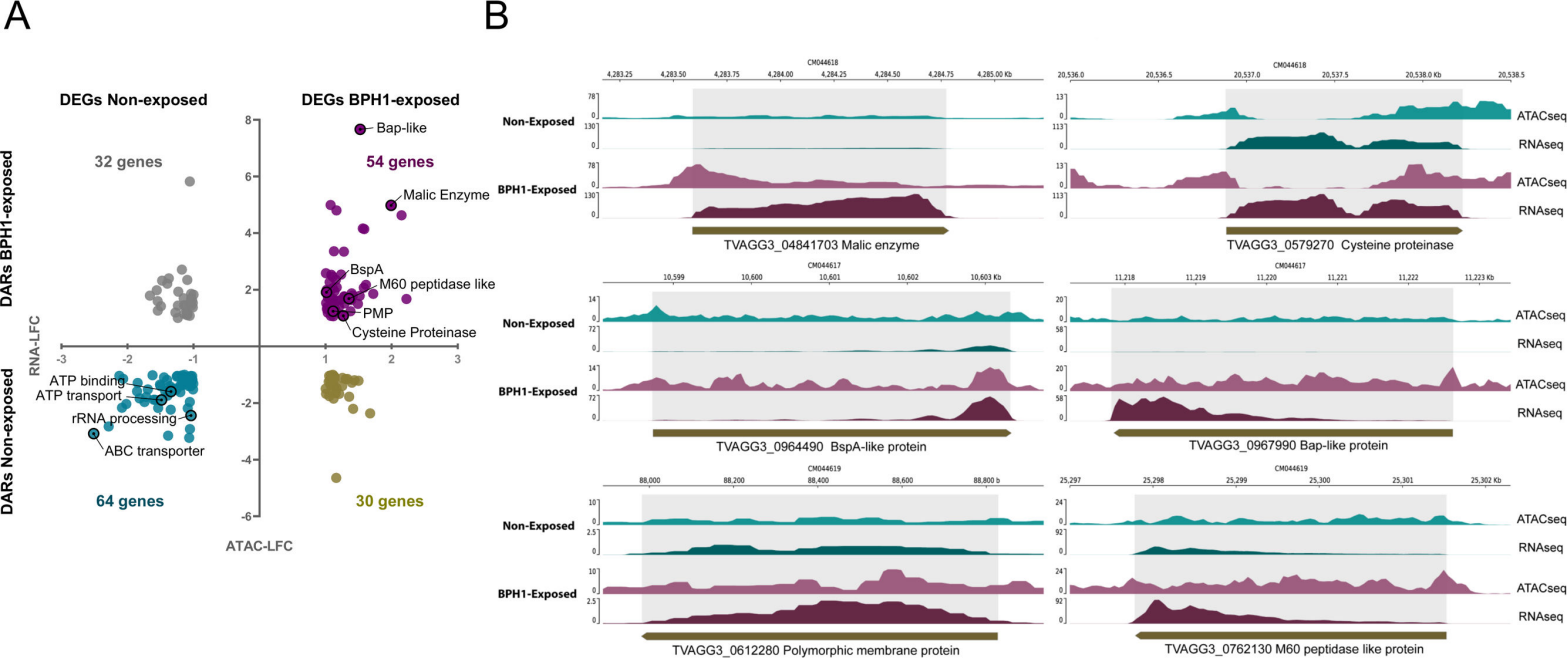
(**A**) Integration of DARs and DEGs in non-exposed and BPH1-exposed parasites. Each point represents a gene, with the *x*-axes indicating changes in gene expression (log2 fold change) and the *y*-axes indicating changes in chromatin accessibility (log2 fold change). A correlation is observed in both directions for the two conditions, with a noticeable difference in the number of genes involved in each quadrant. Upper right quadrant (54 genes, purple): DEGs and DARs in BPH1-exposed parasites. Lower left quadrant (64 genes, cyan): DEGs and DARs in non-exposed parasites. Upper left quadrant (32 genes, gray): DEGs with less accessible chromatin in strain in non-exposed parasites. Lower right quadrant (30 genes, brown): DEGs with less accessible chromatin in BPH1-exposed parasites. (**B**) Comparative chromatin accessibility and gene expression of selected pathogenesis-related genes under non-exposed and BPH1-exposed conditions. ATAC-seq and RNA-seq profiles are shown for six genes of interest (TVAGG3_04841703, malic enzyme; TVAGG3_0967990, Bap-like protein; TVAGG3_0964490, BspA-like protein; TVAGG3_0579270, cysteine proteinase; TVAGG3_0612280, polymorphic membrane protein; and TVAGG3_0762130, M60 peptidase-like protein), illustrating chromatin accessibility and gene expression levels in parasites not exposed to BPH1 cells (non-exposed, cyan) and in parasites exposed to BPH1 cells (BPH1-exposed, purple).

Since chromatin accessibility remains unchanged within 6mA-flanked intervals, we next examined whether changes in other genomic regions could influence transcriptional activation or repression of genes involved in the process of host-parasite interaction. To this end, we analyzed differentially accessible regions (hereafter referred to as DARs) within the genome in BPH1-exposed and non-exposed parasites (|log2 fold change| ≥ 1 and FDR < 0.05). We identified a total of 1,562 statistically significant differentially accessible regions: 775 DARs were identified in non-exposed parasites and 787 DARs in BPH1-exposed parasites (Fig. 7A; Table S6). The distribution of differential ATAC-seq peaks across different genomic areas revealed that 67% of open chromatin regions in non-exposed parasites and 54% in BPH1-exposed parasites are located within gene-coding sequences (named exon) (Fig. 7B). Additionally, 13% of DARs were found in promoter regions, 17% in intergenic regions, and 3% at TTS in non-exposed parasites. In BPH1-exposed parasites, the distribution was 14% in promoter regions, 29% in intergenic regions, and 3% at TTS (Fig. 7B). To explore the functional implications of the observed changes in chromatin accessibility, we performed a GO enrichment analysis on differentially accessible regions identified in free-living parasites and those attached to host cells (Fig. 7C). In free-living parasites, GO analysis indicated that DARs are associated with various metabolic processes, including carbohydrate, amino acid, and sulfur compound metabolism, with a particularly notable enrichment in biological processes linked to multiple metabolic pathways (Fig. 7C). These findings suggest that the parasites sustain diverse metabolic activities, potentially supporting their adaptation and growth in fluctuating environmental conditions in their free-living state. In contrast, in parasites attached to host cells, DARs were enriched in structural and regulatory processes, including telomere organization, cell motility, microtubule-based movement, and transcriptional regulation. This enrichment suggests that host cell attachment triggers changes in chromatin organization, likely reflecting the parasite’s need to modulate gene expression to adapt its behavior and morphology when in contact with host tissues. To further investigate whether the observed chromatin changes are linked to transcriptional modulation, we analyzed the level of expression (FPKM) of genes associated with DARs located within promoter and/or coding regions (Fig. 8A; Table S7). Specifically, we first examined DARs identified in BPH1-exposed parasites and compared the expression of their associated genes between exposed and non-exposed conditions. Among these DARs, 112 genes showed an increase in expression of at least 1.5-fold, while 137 genes exhibited decreased expression upon parasite attachment to host cells (Fig. 8A, left panel). Similarly, analysis of DARs identified in non-exposed parasites revealed that 210 genes displayed a positive correlation between chromatin accessibility and expression, whereas 74 showed an inverse correlation (Fig. 8A, right panel). Gene ontology analysis of the correlated genes revealed distinct biological signatures between the two conditions. In non-exposed parasites, correlated genes were enriched in metabolic processes such as carbohydrate, amino acid, and sulfur compound metabolism, indicating active metabolic remodeling during the free-living state (Fig. 8B). Interestingly, correlated genes in BPH1-exposed parasites were mainly involved in cell adhesion, cytoskeleton organization, and transcriptional regulation (Fig. 8B). These results suggest that chromatin accessibility might regulate genes involved in the process of host cell attachment. To gain further insight into the regulatory features of these correlated genes, we analyzed the promoter sequences of the 112 genes that exhibited both increased chromatin accessibility and expression in the BPH1-exposed condition. Although several motifs were detected, only one showed significant differential enrichment compared with the non-exposed state—an ATTSAAT consensus motif predominantly located upstream of transcription start sites (Fig. 8C). The positional distribution of this motif within −100 to +50 bp of the TSS revealed a strong enrichment upstream of the TSS, peaking around −40 bp (Fig. 8C). This suggests that the ATTSAAT motif may represent a potential cis-regulatory element associated with the transcriptional activation of genes involved in the parasite’s response to host cell contact. To further assess the extent to which changes in chromatin accessibility modulate gene expression, we identified genes containing both DARs and DEGs under free-living and exposed conditions (Table S8). Out of a total of 775 DARs identified in non-exposed parasites (Fig. 7A and B), 64 genes were differentially expressed when parasites are not in contact with host cells (Fig. 9A). Similarly, out of the 787 DARs identified in parasites exposed to BPH1 parasites (Fig. 7A and B), 54 genes differentially increased their expression when the parasites are attached (Fig. 9A). These results suggest that variations in chromatin accessibility may regulate the differential expression only in a specific set of genes. Importantly, increased chromatin accessibility associated with higher gene expression when parasites are exposed to BPH1 cells was found in several genes that have been previously described to modulate parasite adherence and pathogenesis (19). Among the 54 genes with increased expression and chromatin accessibility upon host cell contact, we identified several genes previously described to have a role during the process of *T. vaginalis*-host cell attachment. Specifically, four BspA-like genes (TVAGG3_0536200, TVAGG3_0964490, TVAGG3_1032820, TVAGG3_0361640), two Bap-like genes (TVAGG3_0967990, TVAGG3_0837410), one Pmp-like gene (TVAGG3_0612280), one cysteine proteinase (TVAGG3_0579270), and one peptidase M60 were detected in our analysis (Table S8; Fig. 8B). Although our results indicate that changes in chromatin accessibility correlated with gene expression are relatively limited, chromatin accessibility may still play a critical role in modulating the expression of genes involved in processes that facilitate adhesion and/or cytotoxicity. For example, transcriptional profiling of four genes previously implicated in *T. vaginalis* pathogenesis shows that these genes exhibit low expression and low chromatin accessibility in unexposed parasites but become significantly upregulated and more accessible upon exposure to prostate cells (Fig. 8B). These findings suggest that their activation during parasite-host interaction seems to be associated with increased chromatin accessibility. Alternatively, among the 64 genes that are more expressed and accessible in non-exposed parasite, we identified genes associated with energy metabolism and transport, such as ATP-binding proteins (TVAGG3_0409620, TVAGG3_0573580), proton transporters (TVAGG3_0543700, TVAGG3_0903240, TVAGG3_1040600, TVAGG3_0567620), as well as genes implicated in the regulation of translation and protein biosynthesis (TVAGG3_0320690, TVAGG3_0379900, TVAGG3_0325600) (Fig. S2). The identified genes in this analysis highlight the metabolic and translational processes that are more active in the free-swimming form of the parasite, suggesting a heightened capacity for energy production and protein synthesis, likely contributing to the parasite’s ability to proliferate and survive outside the host environment.

Given that accessible chromatin might also negatively regulate gene expression, potentially by providing access to repressor complexes, we also analyzed the correlation between DARs and silenced genes in free-living parasites as well as parasites exposed to BPH1 cells (Fig. 8A). Specifically, we identified 32 genes more expressed upon *T. vaginalis* exposure to host cells that contain less accessible chromatin compared to the non-exposed parasites. As an example, genes involved in protein ubiquitination (TVAGG3_0885730, TVAGG3_0397910), antioxidant enzymes (TVAGG3_0807190, TVAGG3_0737650), and one cysteine-type peptidase protein (TVAGG3_0148970) demonstrated greater expression paired with lower chromatin accessibility in parasites attached to host cells (Table S8; Fig. 8A). Similarly, we identified 30 genes differentially expressed in the non-exposed parasites with poorly accessible chromatin compared to parasites exposed to BPH1 cells (Fig. 8A). Among them, we identified genes associated with armadillo repeat-containing protein family (TVAGG3_0236840, TVAGG3_0266770, TVAGG3_0489570), genes associated with motility (TVAGG3_0811070, TVAGG3_0558700), or linked to aggrephagy-related functions (TVAGG3_0443890) (Table S8). Together, these findings indicate that the accessibility of chromatin in the promoter region and/or gene body is not always associated with active transcriptional activity, as was recently demonstrated (39).

## DISCUSSION

Despite being a pathogen responsible for a highly prevalent human infection, the molecular mechanisms underlying *T. vaginalis* pathogenesis remain poorly understood. Regulation of gene expression, mainly adherence factors, is key to host attachment (19, 21, 26), yet the mechanisms involved in transcriptional regulation are unclear. Given the increasing evidence of epigenetic control in *T. vaginalis* (21, 30, 33)*,* we investigated here the role of 6mA and chromatin accessibility during host-parasite interaction.

Consistent with the findings of previous studies (26), RNA-seq analysis identified a core set of approximately 24,000 expressed genes with 2,069 upregulated and 1,554 downregulated genes upon exposure to human prostate cells (BPH1). Upregulated genes include several protein families previously implicated in parasite pathogenesis, such as leucine-rich repeat BspA family proteins (19, 20), Bap-like genes (19, 21), and various proteases (22, 40–42), reinforcing their roles in adherence and pathogenesis. The BspA protein family represents the largest gene family encoding potential surface proteins, which are likely to play a key role in modulating *T. vaginalis* attachment to host cells (20, 43). Similarly, members of the Bap-like protein family have been implicated in parasite pathogenesis, as previous studies have shown that members of the Bap-like protein family are differentially expressed in adherent strains, and overexpression of TvBAP1 and TvBAP2 genes in a poorly adherent strain significantly increased parasite attachment to human cervical epithelial cells (19, 21). Furthermore, the expression of TvBAP1 and TvBAP2 has been shown to be regulated epigenetically, particularly through histone acetylation (21). Similarly, evidence for multiple roles for rhomboid intramembrane and subtilisin-like serine proteases in the pathogenesis of divergent protozoan parasites has emerged in recent years. Riestra et al. (22) demonstrated that a rhomboid protease (TvROM1) and its substrate(s) play a role in modulating attachment to and lysis of host cells, key processes in *T. vaginalis* pathogenesis (11, 44). Similarly, an *Entamoeba histolytica* rhomboid protease has been demonstrated to cleave a surface lectin involved in phagocytosis and immune evasion (45). Moreover, multiple rhomboid proteases and subtilisin-like proteases are known to play critical roles in adherence, invasion, and survival of *Toxoplasma* and malaria parasites (46, 47). Several cysteine protease-encoding genes were upregulated during *T. vaginalis* host interaction, supporting their suspected central role regarding virulence, cytoadherence, hemolysis, and cytotoxicity (40, 48, 49). Enrichment analysis identified additional genes linked to transcription, kinase, and phosphatase activity, likely involved in host adaptation. Conversely, downregulated genes were enriched for functions related to ion and RNA binding, as well as ATP-dependent transporter activity, suggesting a potential reallocation of metabolic and cellular resources during host interaction. These findings indicate that *T. vaginalis* orchestrates a dynamic transcriptional response during host interaction, with upregulated genes primarily involved in processes such as adhesion and proteolysis, potentially reflecting the parasite’s adaptive mechanisms for adherence and survival in the host environment.

We also analyzed 6mA patterns in parasites exposed to BPH1 cells. N6-methyladenine, and not 5-methylcytosine, is the primary DNA methylation mark in *T. vaginalis* (33). This mark was predominantly found in intergenic regions, where it is associated with the regulation of gene expression and chromatin architecture (33). Consistent with this, our analysis identified the highest proportion of 6mA peaks at intergenic regions across both conditions. However, slight increases were seen in exons and transcription termination sites. A total of 302 differentially methylated regions were detected when compared to the methylation pattern of parasites unexposed and exposed to host cells. Integration of MeDIP-seq and transcriptomic data did not reveal a consistent correlation between DNA methylation and transcriptional changes, suggesting that 6mA may modulate gene expression in a context-dependent manner. This finding underscores the complexity of the underlying regulatory mechanisms and indicates that 6mA likely acts not as a universal repressor or activator, but rather as a fine-tuning modulator within a multifaceted regulatory landscape. However, we cannot rule out the possibility that 6mA is a dynamic epigenetic mark that may exert its effects at different stages of the host-parasite interaction. Further analysis at additional time points will be necessary to clarify the temporal role of 6mA in gene regulation. To gain deeper insight into the role of 6mA, functional studies targeting parasite-specific DNA 6mA methyltransferases or demethylases are required. Although several putative 6mA DNA methyltransferases have been annotated in the *T. vaginalis* genome database (TrichDB), none have yet been functionally validated. Moreover, no canonical DNA 6mA demethylases have been identified in the *T. vaginalis* genome (36, 50). This absence could reflect the presence of non-conserved demethylating enzymes, the lack of an active 6mA demethylation process, or the operation of an alternative pathway—such as the base excision repair-mediated demethylation mechanism described in plants (51). Identifying the enzymes responsible for regulating 6mA levels in *T. vaginalis* will be key to uncovering the mechanisms and biological functions of this epigenetic modification.

Beyond individual gene regulation, 6mA methylation may also influence higher-order chromatin structure. We identified 6mA-flanked regions associated with either transcriptionally active or repressive intervals in both BPH1-exposed and free-living parasites. Interestingly, regions with more genes were often repressive, while smaller regions tended to be active, suggesting that 6mA spatial organization influences chromatin states. ATAC-seq analysis revealed a strong correlation between chromatin accessibility and gene expression within these regions, supporting a direct link between 6mA and chromatin structure. However, no major differences in the formation or accessibility of 6mA-flanked intervals were observed between exposed and unexposed parasites, indicating that these structures may not be the main drivers of gene regulation at this specific timepoint or in this host cell context. The process of *T. vaginalis*-host interaction likely involves multifactorial mechanisms, potentially including additional factors or combinatory effects beyond these modifications.

Although no major changes in chromatin accessibility were observed within 6mA-flanked intervals, host cell exposure triggered broader changes in chromatin accessibility, particularly at promoters and coding regions. Notably, this change in chromatin accessibility correlated with changes in gene expression for a subset of genes previously linked to host cell adhesion. Specifically, genes such as surface antigen BspA-like, cysteine proteinases, Bap-like and Pmp-like genes, and one peptidase M60 exhibited changes in chromatin accessibility that correlated with changes in gene expression. This highlights the role of chromatin dynamics in regulating genes involved in host interaction.

This study aims to bridge this gap by investigating how chromatin structure and accessibility influence gene expression and contribute to the parasite’s adaptive strategies during host cell attachment. Our study provides a foundation for exploring chromatin-based regulation in parasitic protozoa and underscores the need to investigate additional layers of epigenetic and post-transcriptional control in host-parasite interactions. Understanding the molecular mechanisms that govern the interaction between *Trichomonas vaginalis* and its host is crucial for elucidating the processes that underline parasite adherence and pathogenesis.

## MATERIALS AND METHODS

### Parasites, cell cultures, and media

*Trichomonas vaginalis* strain B7268 (52) was cultured in tubes under microaerophilic conditions at 37°C in TYM medium (tryptose, yeast extract, and maltose) (53) supplemented with 10% horse fetal serum, 10 U/mL penicillin, and 10 µg/mL streptomycin (Invitrogen).

The human benign prostate hyperplasia (BPH-1) cells were cultured in RPMI–10 U/mL penicillin–10 µg/mL streptomycin with 10% fetal bovine serum (all from Invitrogen) as previously described (35). BPH-1 cells were seeded onto tissue culture flasks and allowed to form a confluent monolayer at 37°C in 5% CO_2_. Prior to the co-incubation, BPH-1 cultures and parasites were washed three times in PBS, pH 7.2. Subsequently, BPH-1 cells were co-incubated with *T. vaginalis* at cell ratios of 2:1 parasite:BPH-1 for 90 min. Non-attached parasites were removed by thorough washing. For comparison, parasites in the absence of host cells were analyzed.

### RNA-seq

Total RNA was extracted from ~3.5 × 10^6^ *T. vaginalis* according to the protocol outlined in the illustra RNAspin Mini RNA Isolation Kit (GE Healthcare, UK) following the manufacturer’s instructions. The mRNA libraries were paired-end (100 bp) sequenced with Illumina using TruSeq Stranded mRNA (Macrogen, Inc).

### Bioinformatics analysis of RNA-seq data

After sequencing, ~20 million reads were generated per RNA-seq library. The software FastQC (54) was used for quality control of the sequencing. The adapter sequence content was identified and trimmed using Trimmomatic (55). Then, HISAT2 (56) was used to align the RNA-seq data sets to the G3 2022 reference genome sequence (57), and the results showed an overall alignment rate of >90% for all libraries. In order to quantify the counts, featureCounts (58) was used with default paired-end parameters. Principal component analysis was carried out to evaluate the variation through biological replicates. The expression level of each transcript was quantified as fragments per kilobase of exon per million fragments mapped (FPKM) using StringTie (59). Differential gene expression was explored using the DESeq2 package (60). Only changes where |log2 fold change| ≥ 1 and *P*_adj_ < 0.05 were considered significant. To remove potential human contamination, raw reads from BPH1-exposed samples were first aligned against the human reference genome (hg38, UCSC Genome Browser) (61). Only unmapped reads were retained for downstream analysis.

### MeDIP-seq

Twenty micrograms of purified genomic DNA from *T. vaginalis* strain B7268 (52) as well as B7268 exposed to BPH1 cells was diluted in 400 µL 1× immunoprecipitation (IP) buffer (10 mM Na-phosphate, pH 7, 140 mM NaCl, 0.05% Triton X-100) and fragmented to 100 to 500 bp using a water bath sonicator (amp: 50%, cycle: 30 s on and 30 s off, time: 20 min). The sample was divided into aliquots containing 6 to 7 µg of sonicated DNA and heat-denatured; 1 µg was saved and stored at −20°C to use as input control, and the rest was immunoprecipitated overnight at 4°C using 5 µg of anti-6mA antibody (Abcam) bound to protein A magnetic beads (Invitrogen). After three washes with 1× IP buffer, beads containing bound methylated DNA were resuspended in 250 µL digestion buffer (50 mM Tris, pH 8, 10 mM EDTA, 0.5% sodium dodecyl sulfate) with 100 µg Proteinase K (Roche) and incubated at 65°C for 4 h. Eluted DNA and input control samples were purified using phenol/chloroform/isoamyl alcohol extraction followed by EtOH precipitation. MeDIP samples were sent to Quick Biology Inc. for library preparation and paired-end sequencing. Two independent high-throughput sequencing experiments, each containing DNA from independent immunoprecipitations, were performed.

### Bioinformatics analysis of MeDIP-seq data

After the adaptors were trimmed, alignment of high-quality fastq reads to the G3 2022 reference genome sequence (57) was performed using Bowtie2 (62) with -qc-filter parameter and all other parameters at default settings. The BAM files were used as input for MACS2 (63), which was run with the -g parameter set at 1.8e8, -f BAMPE, and all other parameters at default settings. Input reads were used as a control sample for peak calling. Peaks were then filtered by q value (<0.01) and fold enrichment (>2), and replicates were merged by performing an iterative peak overlapping (64). HOMER2 (65) was used for peak annotation. GO enrichment analysis was performed using the Results Analysis feature at TrichDB (36) with default settings. BAM, BED, and index files were then imported into RStudio (version 4.3.1). DMRs were determined using DiffBind with the parameter “minOverlap = 2” and DESeq2 (within DiffBind) considering log2 fold change |≥1 and FDR < 0.05 as significant (66, 67). Profiles of MeDIP-seq peaks, including density heatmaps and average profiles across the defined intervals in both strains, were generated using the deepTools and the plotHeatmap function in Galaxy Bioinformatics (68). To remove potential human contamination, raw reads from BPH1-exposed samples were first aligned against the human reference genome (hg38, UCSC Genome Browser) (61). Only unmapped reads were retained for downstream analysis.

### ATAC-seq

ATAC-seq assays were performed by Quick Biology, Inc. as previously described (37). Briefly, nuclei isolation was performed from 2 million cells. Then, samples were exposed to the Tn5 transposase (Illumina) for 30 min at 37°C. Tn5 transposase acts as a homodimer to simultaneously fragment the chromatin and insert the necessary adapters for downstream amplification and sequencing. Immediately after incubation with Tn5, the samples were purified using a commercial purification kit. This was followed by a 12-cycle PCR to amplify the DNA fragments and a second round of purification. Finally, the libraries were quantified and sequenced in paired-end read mode using the Illumina HiSeq X platform.

### Bioinformatics analysis of ATAC-seq data

After sequencing, ~20 million reads were generated per ATAC-seq library. For quality control of pairwise sequencing data, FastQC software (54) was used. The adapter sequence content was identified and trimmed using Trimmomatic (55). High-quality adapter-free reads were aligned to the G3 *T. vaginalis* reference genome (57) with Bowtie2 using -X 2000, -3 10 and all other parameters predetermined by the program (62). The resulting BAM files were filtered for quality (>Q10) and used as input files in the MACS2 program, which was run with the parameters -f BED, -g 1.8e8, --shift − 100, and --extsize 200 (63). BAM, BED, and index files were then imported into RStudio (version 4.3.1). DARs were determined using DiffBind with the parameter “minOverlap = 2” and edgeR (within DiffBind) considering log2 fold change | ≥ 1 and FDR < 0.05 as significant (66, 67). The peaks were annotated using the *T. vaginalis* reference genome with HOMER (annotatePeaks.pl) (65). Profiles of ATAC-seq peaks, including density heatmaps and average profiles across the defined intervals in both conditions, were generated using the deepTools and the plotHeatmap function in Galaxy Bioinformatics (68). To remove potential human contamination, raw reads from BPH1-exposed samples were first aligned against the human reference genome (hg38, UCSC Genome Browser) (61). Only unmapped reads were retained for downstream analysis.

### Within-interval expression coherence analysis

To evaluate whether genes located within the same 6mA-flanked interval exhibit coordinated expression patterns, we calculated a coherence score based on binarized FPKM values (expressed if FPKM > 1). For each interval, we determined the proportion of genes sharing the predominant expression state and averaged these proportions across samples to obtain an interval-specific coherence value. To assess whether the observed coherence exceeded random expectation, we generated 1,000 random gene sets for each interval, matching the number of genes per interval but not fixed across all intervals. The mean coherence of these random sets was compared to the observed values using a Wilcoxon rank-sum test implemented in R (v.4.3.1). In parallel, we quantified the similarity in continuous FPKM values among genes within each interval using the ICC, which estimates the proportion of total variance explained by interval membership. The ICC was computed for both exposed and non-exposed conditions and compared against the distribution obtained from 1,000 randomized gene sets of equivalent size. Both analyses were performed exclusively on Active and Repressive intervals, excluding those classified as non-defined.

### Motif enrichment analysis

To identify DNA sequence motifs potentially associated with condition-specific chromatin accessibility, we first performed *de novo* motif discovery using STREME (MEME Suite v.5.5.5) (69). The analysis was conducted on DNA sequences spanning −100 to +50 bp relative to the TSS of genes located within DARs that also showed ≥1.5-fold higher FPKM values in the BPH1-exposed condition (*n* = 112 genes). As background, we used control sequences of identical length randomly sampled from the *T. vaginalis* genome, running STREME in discriminative mode with the parameters --dna --minw 6 --maxw 12 --pvt 0.05 --order 0. Motif enrichment was subsequently assessed using AME (MEME Suite v.5.5.5), by comparing the 121 promoter-associated DARs to the remaining DARs detected in the BPH1-exposed condition that did not display increased expression. This analysis identified the motif ATTSAAT as significantly enriched (*P* = 6.07 × 10^−3^).

Relative motif positions were used to compute a kernel density estimation in R (v.4.4.0) using the density() function with a Gaussian kernel. The resulting position (*x*) and density (*y*) values were exported as a tab-separated file and plotted in GraphPad Prism for Windows version 8.0.2 to visualize the smoothed distribution of motif frequencies across the promoter regions.

### Chromatin accessibility and gene expression differential data

To classify each gene based on its differential expression (DEG) and chromatin accessibility (DAR), RNA-seq and ATAC-seq data sets were integrated. For this purpose, genes were classified as differentially accessible (DAR) if they exhibited a log fold change (log2FC) in chromatin accessibility that met the threshold for significance (|log2-fold change | ≥ 1 and FDR < 0.05). Similarly, genes were classified as differentially expressed (DEG) if their expression levels showed significant changes (|log2 fold change | ≥ 1 and *P*_adj_ < 0.05). Then, to visualize the relationship between DAR and DEG, a scatter plot was created, where each gene was represented by plotting its log2FC in chromatin accessibility on the *y*-axis and log2FC in gene expression on the *x*-axis.

### Gene ontology analysis

The gene ontology enrichment analysis was performed with the Results Analysis option of TrichDB (36) using the default parameters.

## Data Availability

All data supporting the findings of this study are available within the paper and its supplemental information. All sequencing data that support the findings of this study have been deposited in the National Center for Biotechnology Information Sequence Read Archive (SRA) and are accessible through accession number PRJNA1283232.

## SUPPLEMENTAL MATERIAL

Figure S1 mbio.03175-25-s0001.pdf PCA plots.

Figure S2 mbio.03175-25-s0002.pdf Accessibility and expression changes.

Table S1 mbio.03175-25-s0003.xlsx Differential expression genes.

Table S2 mbio.03175-25-s0004.xlsx m6A MedIP-seq peaks.

Table S3 mbio.03175-25-s0005.xlsx Differential methylated regions.

Table S4 mbio.03175-25-s0006.xlsx 6mA flanked intervals.

Table S5 mbio.03175-25-s0007.xlsx Accessible chromatin regions.

Table S6 mbio.03175-25-s0008.xlsx Different accessible regions.

Table S7 mbio.03175-25-s0009.xlsx DAR expression level.

Table S8 mbio.03175-25-s0010.xlsx DAR DEG.

ASM does not own the copyrights to Supplemental Material that may be linked to, or accessed through, an article. The authors have granted ASM a non-exclusive, world-wide license to publish the Supplemental Material files. Please contact the corresponding author directly for reuse.
