# Supplementary material for: Influence of DNA methylation and chromatin accessibility on regulation of gene expression during Trichomonas vaginalis-host cell interaction
Source: mBio. 2025 Dec 3;17(1):e03175-25. doi: 10.1128/mbio.03175-25 (PMC12802312; doi:10.1128/mbio.03175-25)
Supplement: Figure S1 — PCA plots. [file mbio.03175-25-s0001.pdf]

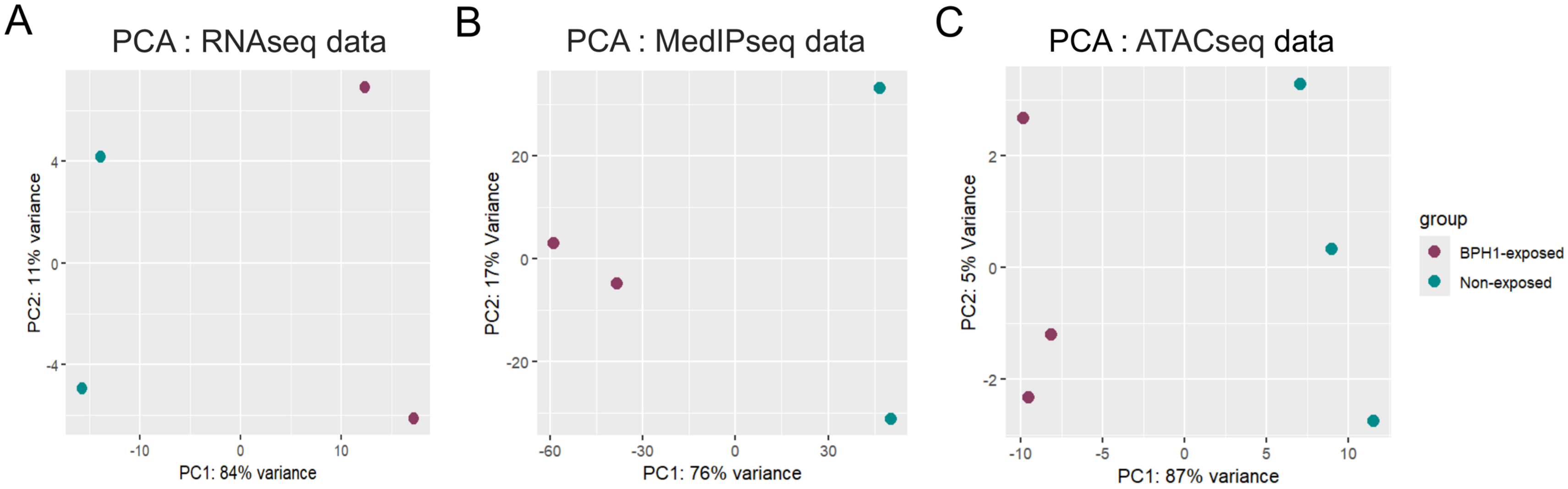

Supplementary Figure 1. A. Principal Component Analysis (PCA) plot from RNA-seq data of non-exposed (cyan) and BPH1-exposed (magenta) parasites. The first principal component (PC1) explains 84% of the variance, separating the two conditions, while the second principal component (PC2) accounts for 11% of the variance. Data points represent individual biological replicates. **B.** Principal Component Analysis (PCA) plot of 6mA profiles obtained from MedIP-seq of non-exposed (purple) and BPH1-exposed (cyan) parasites. The first principal component (PC1) explains 92% of the variance, separating the two conditions, while the second principal component (PC2) accounts for 7% of the variance. Data points represent individual biological replicates. **C.** Principal Component Analysis (PCA) plot of chromatin accessibility profiles obtained from ATAC-seq of non-exposed (purple) and BPH1-exposed (cyan) parasites. The first principal component (PC1) explains 87% of the variance, separating the two conditions, while the second principal component (PC2) accounts for 5% of the variance. Data points represent individual biological replicates.
