# Supplementary material for: Influence of DNA methylation and chromatin accessibility on regulation of gene expression during Trichomonas vaginalis-host cell interaction
Source: mBio. 2025 Dec 3;17(1):e03175-25. doi: 10.1128/mbio.03175-25 (PMC12802312; doi:10.1128/mbio.03175-25)
Supplement: Figure S2 — Accessibility and expression changes. [file mbio.03175-25-s0002.pdf]

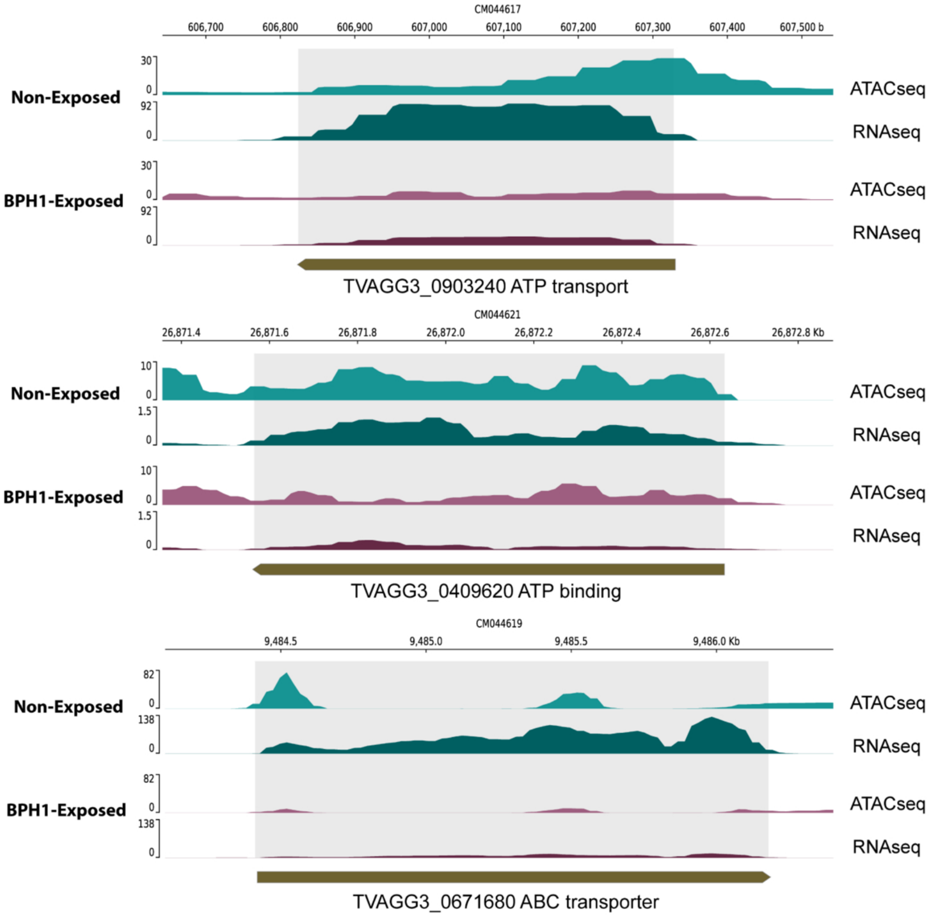

Supplementary Figure 2. Comparative chromatin accessibility and gene expression of genes showing correlated accessibility and expression changes under non-exposed condition. ATAC-seq and RNA-seq profiles are shown for three genes of interest (*TVAGG3_0903240,* ATP transport; *TVAGG3_0409620,* ATP binding; *TVAGG3_0671680*, ABC transporter), illustrating chromatin accessibility and gene expression levels in parasites not exposed to BPH1 cells (Non-exposed, cyan) and in parasites exposed to BPH1 cells (BPH1-exposed, purple).
